# Focus expansion and stability of the spread parameter estimate of the power law model for dispersal gradients

**DOI:** 10.7717/peerj.3465

**Published:** 2017-06-20

**Authors:** Peter S. Ojiambo, David H. Gent, Lucky K. Mehra, David Christie, Roger Magarey

**Affiliations:** 1 Center for Integrated Fungal Reserach, Department of Entomology and Plant Pathology, North Carolina State University, Raleigh, NC, USA; 2 U.S. Department of Agriculture-Agricultural Research Service, Forage Seed and Cereal Reserach Unit, Department of Botany and Plant Pathology, Oregon State University, Corvallis, OR, USA; 3 Center for Integrated Pest Management, North Carolina State University, Raleigh, NC, USA

**Keywords:** Inverse power law, Dispersive waves, Epidemic spread, Parameter stability, Long-distance dispersal

## Abstract

Empirical and mechanistic modeling indicate that pathogens transmitted via aerially dispersed inoculum follow a power law, resulting in dispersive epidemic waves. The spread parameter (*b*) of the power law model, which is an indicator of the distance of the epidemic wave front from an initial focus per unit time, has been found to be approximately 2 for several animal and plant diseases over a wide range of spatial scales under conditions favorable for disease spread. Although disease spread and epidemic expansion can be influenced by several factors, the stability of the parameter *b* over multiple epidemic years has not been determined. Additionally, the size of the initial epidemic area is expected to be strongly related to the final epidemic extent for epidemics, but the stability of this relationship is also not well established. Here, empirical data of cucurbit downy mildew epidemics collected from 2008 to 2014 were analyzed using a spatio-temporal model of disease spread that incorporates logistic growth in time with a power law function for dispersal. Final epidemic extent ranged from 4.16 ×10^8^ km^2^ in 2012 to 6.44 ×10^8^ km^2^ in 2009. Current epidemic extent became significantly associated (*P* < 0.0332; 0.56 < *R*^2^ < 0.99) with final epidemic area beginning near the end of April, with the association increasing monotonically to 1.0 by the end of the epidemic season in July. The position of the epidemic wave-front became exponentially more distant with time, and epidemic velocity increased linearly with distance. Slopes from the temporal and spatial regression models varied with about a 2.5-fold range across epidemic years. Estimates of *b* varied substantially ranging from 1.51 to 4.16 across epidemic years. We observed a significant *b* ×time (or distance) interaction (*P* < 0.05) for epidemic years where data were well described by the power law model. These results suggest that the spread parameter *b* may not be stable over multiple epidemic years. However, *b* ≈ 2 may be considered the lower limit of the distance traveled by epidemic wave-fronts for aerially transmitted pathogens that follow a power law dispersal function.

## Introduction

The spread of new or invasive plant and animal pathogens on a continental scale has important policy implications with regard to the management of disease epidemics since invading species often negatively impact ecosystem function (Brown & Hovmøller, 2002; Crowl et al., 2008; Murray et al., 2011). Quarantine, containment and eradication programs are usually implemented to contain these invasions. In certain circumstances, specific measures may also be developed to prevent potential incursions. Thus, understanding the processes and factors that affect biological invasions on a landscape scale is important to establish and predict the risk of biological invasions. Such an understanding can facilitate planning and designing effective measures to eradicate and contain biological invasions (Zadoks & Van den Bosch, 1994).

A fundamental component in the dynamics of epidemic expansion in both space and time is the contact distribution. The contact distribution defines the likelihood of contact between an infected host at one location and a host or vector at another location. Thus, the contact distribution characterizes the probability of an infectious unit originating from the source coming into contact with a host at some distance away from the source (Aylor, 2003; Madden, Hughes & Van den Bosch, 2007). If the number of healthy, susceptible host individuals is non-limiting, the dynamics of a disease epidemic in space and time depend only on the contact distribution and such dynamics can be characterized as (Madden, Hughes & Van den Bosch, 2007):

(1) \begin{eqnarray*}{Y}_{n+1}(s)={R}_{0}\int \nolimits \nolimits _{-\infty }^{\infty }{Y}_{n}(\xi )\cdot D(s-\xi )d\xi \end{eqnarray*}

where *Y*_*n*+1_(*s*) is the density of infectious individuals at positon *s*, *n* is the number of generations at all possible locations *ξ*, *R*_0_ is the basic reproduction number, *Y*_*n*_(*ξ*) is the density of infectious individual at location *ξ* at generation *n*, and *D*(*s* − *ξ*) is the contact distribution. The contact distribution *D*(*s* − *ξ*), herein denoted for simplicity as *D*(*s*), is a two-dimensional dispersal function, which can be interpreted as the probability that an infectious unit originating at location *ξ* comes into contact with a host at position *s*. All the variables and parameters used in this study are listed and defined in Table 1.

**Table 1:** List of symbols used in this study.

Symbol	Definition	Units^a^
*b*	Exponent (or spread parameter) in the power law model	ND
*D*(*s*)	Contact distribution	m^−2^
$\tilde {D}(s)$	Marginal distribution of *D*(*s* − *ξ*)	m^−1^
*D*(*s* − *ξ*)	Contact distribution, i.e., two dimensional distribution of inoculum produced by individual at position *ξ*	m^−2^
*n*	Number of generations	T
*ξ*	Source position	ND
*σ*^2^	Variance of the contact distribution	m^2^
*R*_0_	Basic reproduction number, i.e., ratio of total number of daughter lesions per original mother lesions	ND
*r*	Vanderplank’s logistic rate parameter	t^−1^
*s*	Target position where epidemic is evaluated	ND
*s*_*t*_	Distance of epidemic wave-front from initial focus at time *t*	m
*s*′	Distance	m
*s*_0_	Initial focus distance	m
*t*	Time	T
*v*	Velocity (or average rate of epidemic expansion)	m t^−1^
*v*_*t*_	Instantaneous velocity at time *t*	m t^−1^
*Y*	Density of infectious individuals	N m^−2^
*Y*_0_	Initial density of infectious individuals	N m^−2^
*Y*′	Pre-specified density of infectious individuals	N m^−2^
*y*(*t*, *s*)	Density of infectious individuals at time *t* and position *s*	N m^−2^
*λ*	Distance parameter in the power law distribution	m
*τ*	Slope of the of spatial regression model	ND
*ψ*	Slope of the temporal regression model	ND

**Notes.**

a ND, dimensionless quantity; *N*, number of individuals; and m, distance.

The velocity of focus expansion is linearly related to the standard deviation of *D*(*s*) and thus, the functional form of *D*(*s*) strongly affects the dynamics of disease epidemics in space and time (Minogue & Fry, 1983; Hastings et al., 2005; Van den Bosch, Zadoks & Metz, 1988). If *D*(*s*) is exponentially bound, then the variance of *D*(*s*) is given as:

(2) \begin{eqnarray*}{\sigma }^{2}=\int \nolimits \nolimits _{0}^{\infty }{s}^{2}\tilde {D}(s)ds\end{eqnarray*}

where $\tilde {D}(s)$ is the marginal contact distribution. In Eq. (2), *σ*^2^ is well defined (i.e., *σ*^2^ ≠ ∞) and solutions to  Eq. (1) will follow a Gaussian distribution resulting in a velocity of focus expansion (*ν*) that is constant, i.e., $\nu =\sigma \sqrt{2\ln ({R}_{0})}$ (Madden, Hughes & Van den Bosch, 2007), where *σ* is the standard deviation of *D*(*s*). This constant velocity of epidemic expansion is characteristic of a travelling wave epidemic. However, if *D*(*s*) has a power law tail, i.e., *D*(*s*) ∝ *s*^−*b*^, where the exponent *b* is the spread parameter, then the variance of *D*(*s*) is a divergent quantity and the solution to Eq. (1) follows a Lévy distribution with power law tails (Reynolds, 2011). In this case, the average rate (or velocity) of epidemic expansion for large times (or generations) approaches (Madden, Hughes & Van den Bosch, 2007; Reynolds, 2011):

(3) \begin{eqnarray*}\nu = \frac{{s}^{{^{\prime}}}}{n} \cong \frac{\lambda }{n} \cdot { \left( \frac{b{Y}_{0}}{2\lambda {Y}^{{^{\prime}}}} \right) }^{ \frac{1}{b} }\cdot \exp \nolimits \left( \frac{n}{b} \ln \nolimits {R}_{0} \right) \end{eqnarray*}

where *s*′ is the distance, *n* is the generation time, *Y*_0_ is the initial density of infected individuals, *Y*′ is a pre-specified density of infected individuals and λ is the distance parameter in the power law distribution. Thus, *v* in Eq. (3) increases exponentially with each successive pair of generations, which is characteristic of a dispersive wave epidemic with accelerating velocity.

Empirical evidence and mechanistic modeling indicate that aerially transmitted pathogens result in epidemics that derive from *D*(*s*) that are non-exponentially bound and follow a power law resulting in dispersive epidemic waves (Ferrandino, 1993; Mundt et al., 2009a; Scherm, 1996). The power law has an interesting mathematical property of being scale-invariant (Gisiger, 2001; Newman, 2005) making it capable of describing dynamics of systems in space and time in a predictable manner. Thus, irrespective of the pathogen, contact distributions that result in dispersive waves are expected to have power law tails with a spread parameter *b*, which can be measured empirically. A formulation of Eq. (1) that incorporates a logistic function for disease increase in time and a power law to describe *D*(*s*) can be written as (Jeger, 1983; Madden, Hughes & Van den Bosch, 2007):

(4) \begin{eqnarray*}y(t,s)= \left\{ \begin{array}{@{}l@{}} \displaystyle \partial y/\partial t=ry(1-y) \\ \displaystyle \partial y/\partial s=by(1-y)/s \end{array} \right. \end{eqnarray*}

where *y*(*t*, *s*) is the disease density at position *s* and time *t*, and *r* is the intrinsic infection rate. Equation (4) provides a simple analytical approach to empirically measure the parameter *b* for dispersive epidemic waves. Assuming isotropic dispersal, partial derivatives of *y*(*t*, *s*) yield an instantaneous measure of the epidemic velocity (*v*_*t*_):

(5) \begin{eqnarray*}{\nu }_{t}= \frac{\partial s}{\partial t} = \frac{\partial s}{\partial y} \times \frac{\partial y}{\partial t} = \frac{r{s}_{t}}{b} \end{eqnarray*}

in which *s*_*t*_ is the distance of the wave-front at time *t* from an initial point source, *s*_0_. In Eq. (5), *ν*_*t*_ is the rate at which the disease focus expands and a plot of *ν*_*t*_ versus distance will result in a straight line with a slope of *r*∕*b* if epidemics start at the same initial distance from the focus, *s*_0_.

A main assumption in Eq. (5) is that all epidemics are first observed at the same *s*_0_. However, a more probable setting of practical interest under field conditions is when epidemics of different rates simultaneously start from their respective sources, which results in *s*_0_ being greater for a faster epidemic. In this case, *s*_0_ is proportional to *r* and time can be expressed using system-specific units of 1∕*r*. Thus, a scaled version of Eq. (5) that is useful to compare different epidemics, can be rewritten as (Ferrandino, 1993; Madden, Hughes & Van den Bosch, 2007; Mundt et al., 2009a; Mundt et al., 2009b):

(6) \begin{eqnarray*}{\nu }_{t}={s}_{t}/b\end{eqnarray*}

In Eq. (6), *ν*_*t*_ for different epidemics will fall on a line with a slope of *b* irrespective of *r* but a faster epidemic will have a greater *s*_0_ and would reach any distance much faster than a slower epidemic (Mundt et al., 2009a; Mundt et al., 2009b). Analytically, Eq. (6) can be used to estimate the value of *b* using a spatial regression approach (Madden, Hughes & Van den Bosch, 2007; Mundt et al., 2009a; Mundt et al., 2009b).

The value of *b* can also be estimated separately using a temporal regression approach. Over time, *s*_*t*_ becomes exponentially more distant from *s*_0_ (Madden, Hughes & Van den Bosch, 2007) and using one unit time steps, the position of the wave-front can be described as (Mundt et al., 2009a; Mundt et al., 2009b):

(7) \begin{eqnarray*}{s}_{t}={s}_{0}{ \left( 1+ \frac{b}{b-1} \right) }^{t}={s}_{0}{ \left( \frac{b}{b-1} \right) }^{t}.\end{eqnarray*}

Equation (7) can be linearized as:

(8) \begin{eqnarray*}\ln \nolimits ({s}_{t})=\ln \nolimits ({s}_{0})+t\ln \nolimits \left( \frac{b}{b-1} \right) .\end{eqnarray*}

The parameter *b* can subsequently be estimated using the temporal regression approach from Eq. (8) based on the slope of the regression of ln(*s*_*t*_) on *t*.

The scale-invariance hypothesis of the power law was tested by Mundt et al. (2009b) using epidemics of plant and animal diseases. Based on epidemics recorded in a single year, Mundt et al. (2009b) observed that the spread parameter was fairly constant regardless of the epidemic scale with *b* ≈ 2, consistent with the lower bound for biologically-realistic asymptotic growth in a square lattice (Chatterjee & Dey, 2016). Factors that influence epidemic expansion may vary from year to year, which may influence the stability of empirical estimates of *b*. While the findings by Mundt et al. (2009b) facilitate prediction of the distance travelled by epidemics fronts of dispersive waves, inaccurate values of *b* could also lead to incorrect predictions and policies for containing invasions.

In addition, when *D*(*s*) follows the power law, *ν*_*t*_ is multiplicatively related to *s*_0_ (Madden, Hughes & Van den Bosch, 2007) and the size of the initial epidemic area is expected to be strongly related to the final epidemic extent for epidemics (Mundt et al., 2013). Although environmental conditions, data collection and processing and spatial variation can influence the expansion of epidemics (Riley, 2007), the stability of the effect of *s*_0_ on the extent of the epidemic spread under factors that can influence epidemic expansion over multiple years has not been determined.

Multiple realizations of an epidemic over time or space are necessary to quantify the stability of parameter estimates for *b* and *s*_0_ on the extent of the epidemic spread. At the landscape scale, obligate biotrophic plant pathogens are excellent model systems to assess the stability of these parameters and processes because dispersal, pathogen incursion, and epidemic development occur annually as host tissue becomes dormant or removed each season (Mundt et al., 2013). Further, ethical issues that would preclude release of human and animal pathogens do not exist with plant pathogens. In this study, a motivating example is found with downy mildew of cucurbits, caused by the oomycete *Pseudoperonospora cubensis*, an economically important disease that affects plants within the family Cucurbitaceae (Ojiambo et al., 2015). The pathogen exhibits significant long distance dispersal at the continental scale (Ojiambo & Holmes, 2011; Ojiambo & Kang, 2013), and since its hosts are sensitive to frost, incursions of the pathogen into northern latitudes from subtropical overwintering areas occur annually (Ojiambo et al., 2015). Ojiambo et al. (2015) presented preliminary data that suggested that epidemic extent may be related to the size of the initial outbreak. Thus, this study utilized cucurbit downy mildew (CDM) as a model system to: (i) establish the consistency in relationship between the size of the initial disease focus and the final extent of the epidemic in multiple epidemic years and (ii) determine the stability of the spread parameter *b* of the power law model over multiple epidemic years in predicting the distance traveled by epidemic wave fronts.

## Materials and Methods

### Data source

Epidemics of CDM recorded in the United States from 2008 to 2014 were analyzed in this study using data obtained from the CDM ipmPIPE program (Ojiambo et al., 2011). The CDM ipmPIPE is part of the United States Department of Agriculture Pest Information Platform for Extension and Education (PIPE) program. Records of confirmed outbreaks of CDM were collected as part of the sentinel and non-sentinel plot monitoring network designed to alert growers on the spread of CDM. Sentinel plots were fixed plots, planted early and designated for weekly surveillance, while non-sentinel plots consisted of commercial fields, research plots, and home gardens. The number of sentinel plots in 2008, 2009, 2010, 2011, 2012, 2013, and 2014, was 84, 85, 48, 44, 54, 48, and 54, respectively, while the number of counties affected by CDM during this period ranged from 41 to 107 (Table 2). The customized section of the CDM ipmPIPE website was used to generate the latitude and longitude data associated with the location of each reported outbreak. Where no geo-referenced data were available, latitude and longitude corresponding to the county centroid (extracted from US Census Bureau 1990 Gazetteer Files) were used as the approximate georeferenced points. The CDM pathogen is not known to overwinter outdoors in the continental United States north of 30 degree latitude and disease spread relies on the annual introduction of the pathogen from overwintering sources in the southern states (Ojiambo et al., 2015). These annual extinction-colonization cycles of the pathogen provides a useful framework to examine focus expansion and velocity of epidemic spread over multiple years.

### Initial focus and epidemic extent

**Figure 1:**
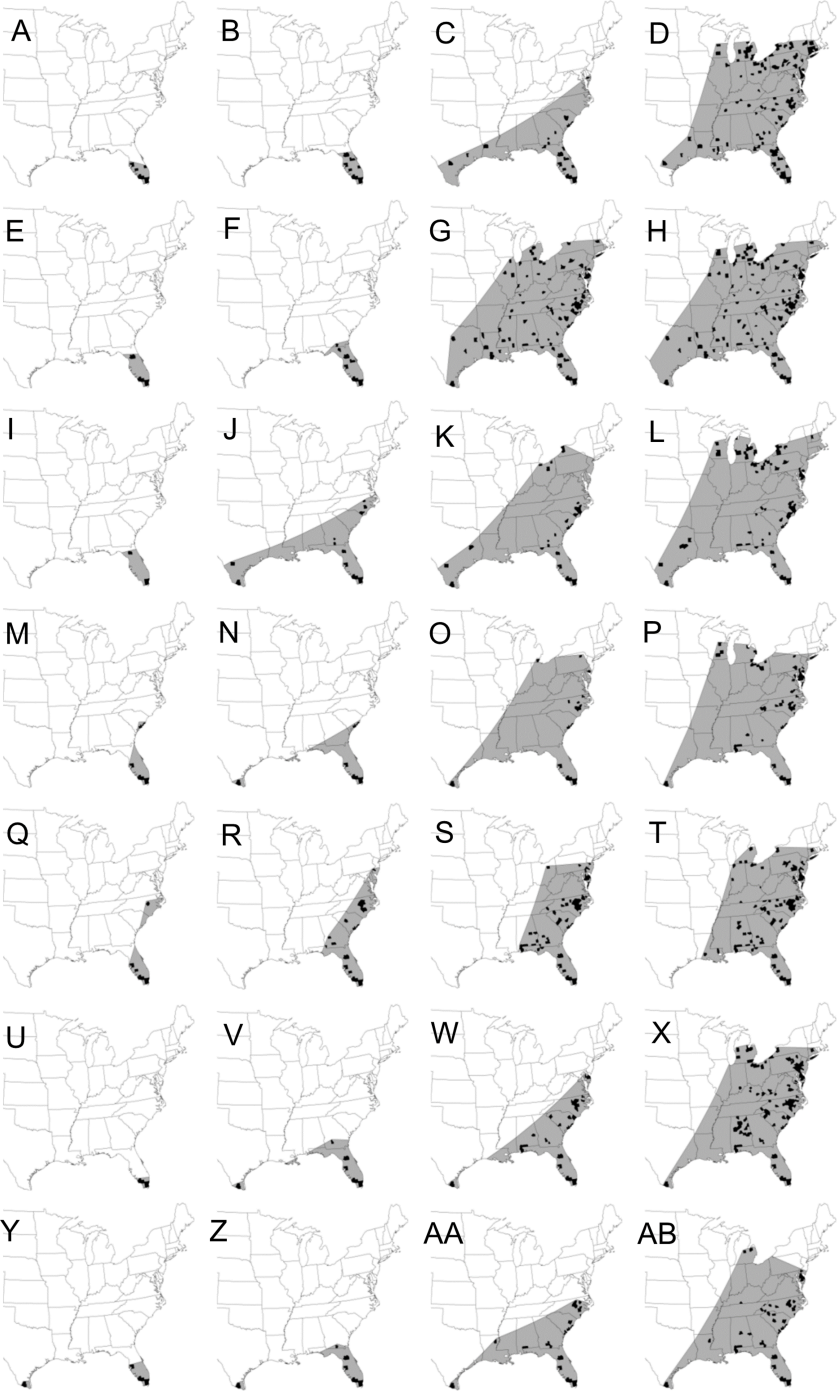
Maps showing the spatial spread of the epidemic wave-front of cucurbit downy mildew in the eastern United States. Maps depicting the continental spread of cucurbit downy mildew across the eastern United States based on cumulative county-level reports of new outbreaks (black fill). The area of epidemic extent (gray shade) is calculated by connecting outbreaks at the leading edges of the epidemic wave-front with a straight line. Epidemic years are presented consecutively in rows with the first row being 2008 and the last row being 2014. For each year, maps are for the epidemic on calendar week 16 in April (first column: A, E, I, M, Q, U, Y), week 20 in May (second column: B, F, J, N, R, V, Z), week 24 in June (third column: C, G, K, O, S, W, AA) and week 28 in July (fourth column: D, H, L, P, T, X, AB). Epidemic maps for 2008 and 2009 were published as preliminary data in Ojiambo et al. (2015).

In each epidemic year, disease prevalence was calculated by expressing the cumulative number of counties with disease reports at each time point as a proportion of the final number of counties with disease outbreaks. Following the methods of Mundt et al. (2013) to determine the effect of initial disease focus on the final extent of the epidemic, weekly maps were constructed to calculate the cumulative extent of epidemic spread based on counties where CDM was reported on any cucurbit host type from 23 March to 31 July. To construct isopleths (i.e., epidemic wave-fronts at time, *t*) of positive detections, counties at the leading edge of the epidemic in each week were connected with a straight line if the resulting angle was convex (Fig. 1). Preliminary maps depicting spread of the epidemic wave-front have been published elsewhere (Ojiambo et al., 2015). Polygon land areas within isopleths were filled-in using the ‘create feature/polygon’ tool in ArcMap version 10.3.1 (ESRI, 2007) to generate maps of epidemic extent. The resulting area of epidemic extent maps was then calculated using Hawth’s Analysis Tools for ArcGIS extension. The relationship between final epidemic areas calculated on July 31 and epidemic areas calculated biweekly in all seven epidemic years were analyzed by linear regression using PROC REG in SAS (version 9.4; SAS Institute, Cary, NC, USA). Pearson correlation analysis was conducted using PROC CORR in SAS to determine whether the number of disease reports per year was associated with the final epidemic area in the same year.

### Focus expansion and spread parameter *b*

In all epidemic maps generated above, 10 equally spaced geographical coordinates were located on each isopleth, and these coordinates were connected to the source location to draw 10 transects. The length of each transect was calculated using ‘GEODIST’ function in SAS (version 9.4; SAS Institute, Cary, NC, USA), which calculates geodetic distance between two location coordinates. The mean length of the 10 transects was the estimate of the distance (*s*_*t*_) traveled by the epidemic in a particular time. The spread parameter *b* was estimated based on *s*_*t*_ using temporal and spatial regression models to quantify the stability of *b* across multiple epidemic years.

In the temporal regression approach, Eq. (6) was expressed using a natural log transformation to allow for the estimation of *b*:

(9) \begin{eqnarray*}\ln \nolimits ({s}_{t})=\ln \nolimits ({s}_{0})+t\cdot \ln \nolimits [b/(b-1)].\end{eqnarray*}

In Eq. (9), the natural log of distance traveled by epidemic, ln(*s*_*t*_), was regressed on time *t* based on least square regression using PROC REG in SAS. The parameter *b* was then estimated from the slope (*τ*) of Eq. (9) as: *b* = e^*τ*^∕(e^*τ*^ − 1). In the spatial regression approach, the instantaneous velocity, *ν*_*t*_, estimated using Eq. (5) was regressed against *s*_*t*_ for each time step using PROC REG in SAS. Similarly, the inverse of the slope (*ψ*) of the resulting regression of *ν*_*t*_ on *s*_*t*_ provided an estimate of the spread parameter as: *b* = *ψ*^−1^.

Covariance analysis was conducted to determine the stability of the spread parameter *b* estimated from temporal and spatial regression models for all epidemic years. The analysis was performed using PROC GLM in SAS with *b* and year as factors. A non-significant *b* × year interaction would suggest the power law spread parameter is fairly stable over multiple epidemic years.

## Results

### Initial focus and epidemic extent

**Figure 2:**
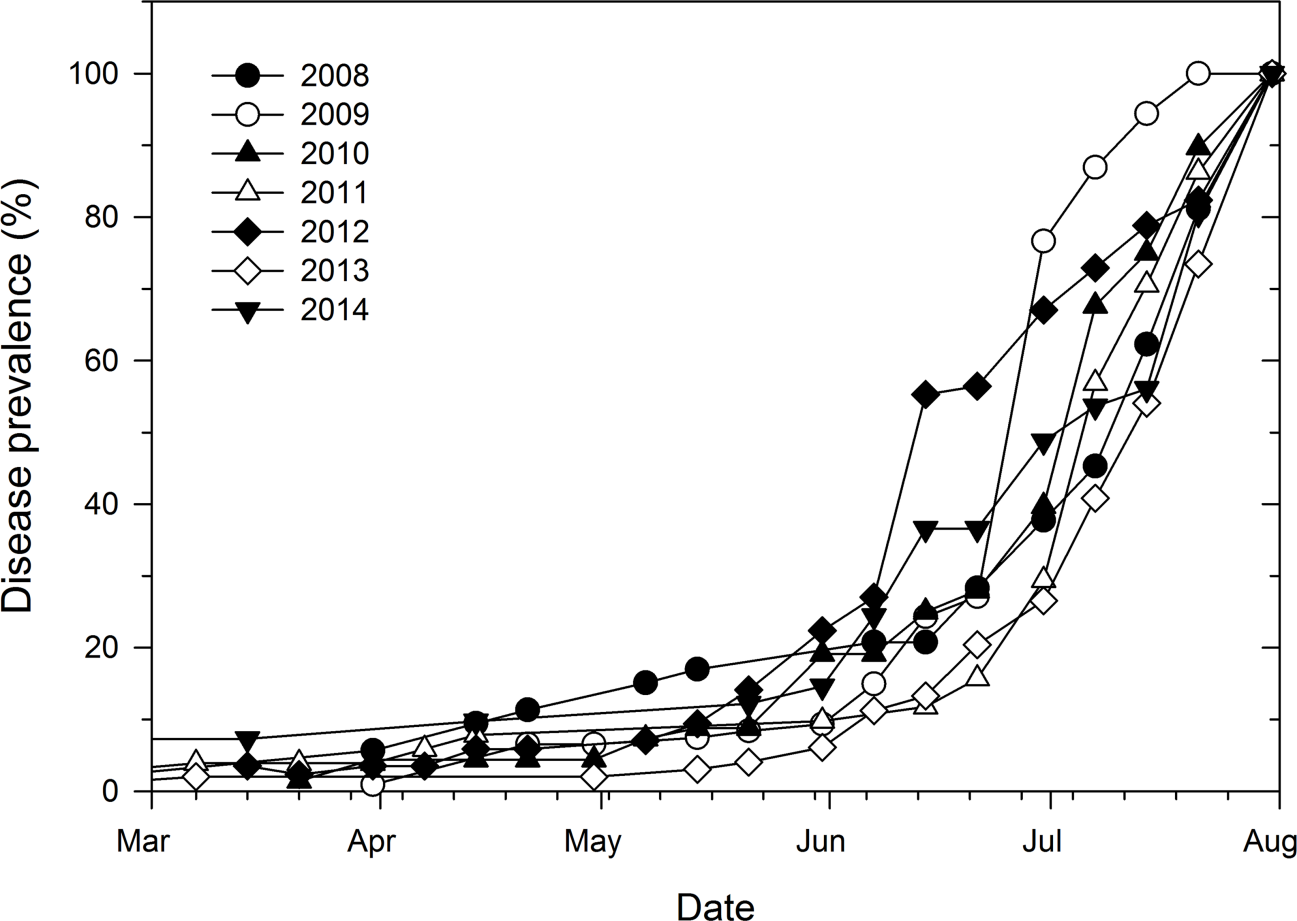
Temporal prevalence of cucurbit downy mildew in the eastern United States based on county-level reports of disease outbreaks. Prevalence of cucurbit downy mildew in the eastern United States based on cumulative number of counties with disease reports as a proportion of the final number of counties with disease outbreaks in each epidemic year.

The increase in disease prevalence over time was characteristic of a sigmoid curve with prevalence being generally low and constant (2 to 16% across epidemic years) from March 1 to May 14 (Fig. 2). Disease prevalence increased exponentially thereafter which was followed by a gradual decrease in the number of new cases towards the end of the monitoring period especially in 2009. In addition, the increase in disease prevalence during the exponential phase from June 7 to June 30 was more rapid in 2012 and 2009 than the corresponding increase in all the other epidemic years (Fig. 2).

Final epidemic extent varied across epidemic years (Fig. 1) with a 1.5-fold difference in final epidemic extent for the epidemic year with the least disease spread and that with the most disease spread (Table 2). The least epidemic spread was observed in 2012 with an area of 4.16 × 10^8^ km^2^, while the epidemic year 2009 had the most disease spread with a final extent of 6.44 × 10^8^ km^2^. Final epidemic spread was similar for epidemic years 2009 and 2010. Similarities in epidemic spread were also observed for epidemic years 2012 and 2014, although the epidemic extent was much lower than that observed in 2009 or 2010 (Table 2). The number of disease reports varied considerably among epidemic years ranging from 71 disease cases in 2014 to 183 cases in 2012 (Table 2). In addition, Pearson correlation coefficient showed that the number of disease reports per year was not significantly (*r* = 0.15; *P* = 0.7414) associated with final epidemic extent in the same year. Similarly, no significant (*r* = 0.22; *P* = 0.6283) association was observed between the number of sentinel plots and the corresponding final epidemic extent.

**Table 2:** Cases of cucurbit downy mildew and number of counties with reported cases in the Cucurbit Integrated Pest Management Pest Information for Extension and Education project database and the final epidemic area in the week ending July 31 in each epidemic year.

Year	Number of states affected	Number of counties affected	Number of disease reports	Final epidemic area (×10^8^ km^2^)
2008	15	53	102	4.96
2009	22	107	175	6.44
2010	15	68	141	6.32
2011	15	51	92	5.53
2012	17	85	183	4.16
2013	18	98	182	4.94
2014	13	41	71	4.41

**Figure 3:**
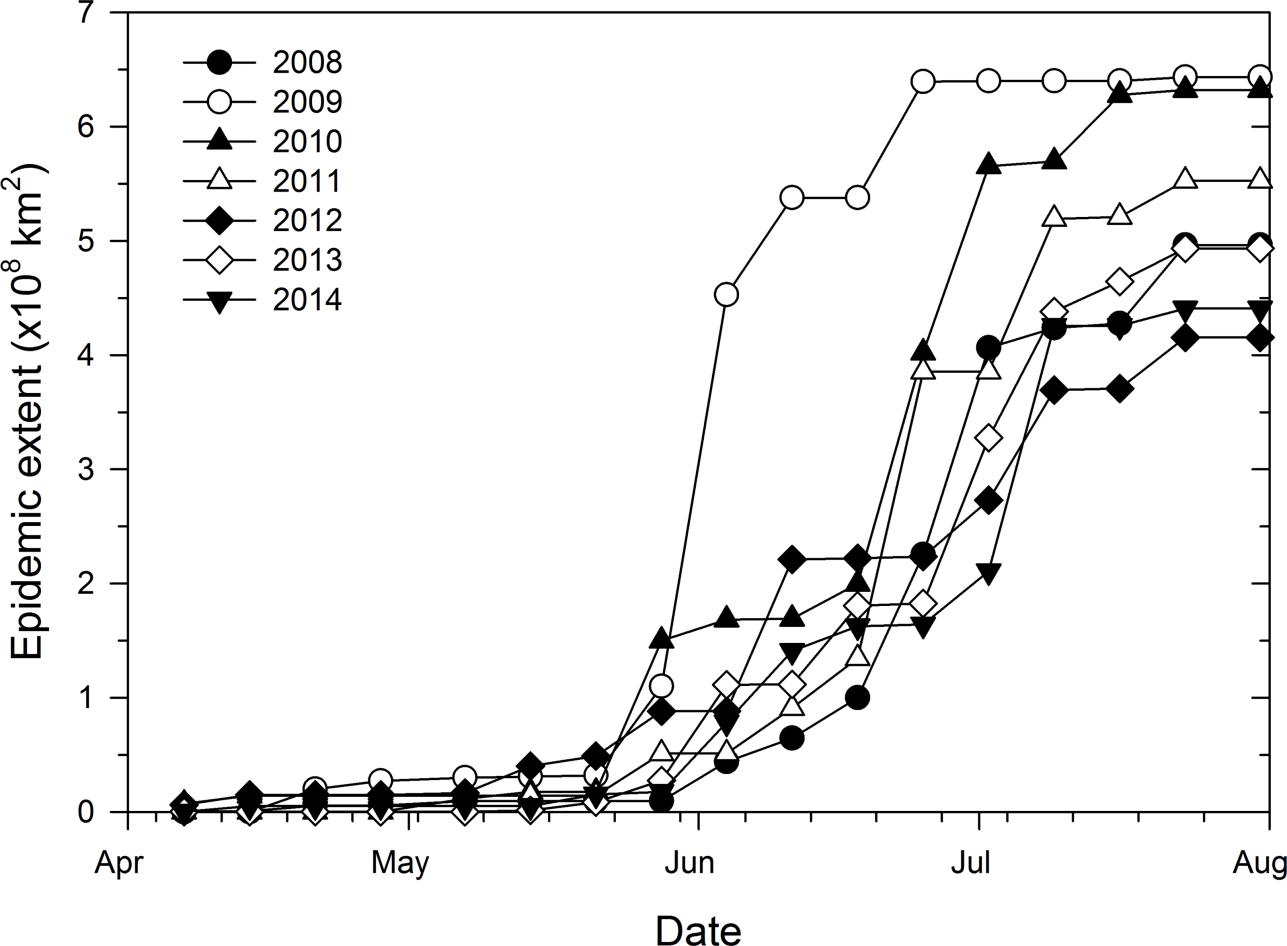
Progress of epidemic area of cucurbit downy mildew based county-level reports of disease outbreaks. Temporal progress of weekly epidemic area of cucurbit downy mildew in the eastern United States based on county-level cumulative reports of new disease outbreaks.

Temporal increase of weekly epidemic area was also characteristic of a sigmoid curve with epidemic areas being very small and relatively uniform from April 7 to May 21 (Fig. 3). Thereafter, epidemic areas increased exponentially before leveling-off between June 25 and July 23, depending on the epidemic year (Fig. 3). In addition, weekly epidemic areas reflected final epidemic areas as early as June 4 whereby the epidemic year 2009, which had the highest final epidemic, also had the highest weekly epidemic area compared to all the other years (Fig. 3). For the remaining epidemic years, consistent ranking of weekly epidemic areas with final epidemic extent was more apparent from July 2 until the end of disease monitoring period (Fig. 3).

**Figure 4:**
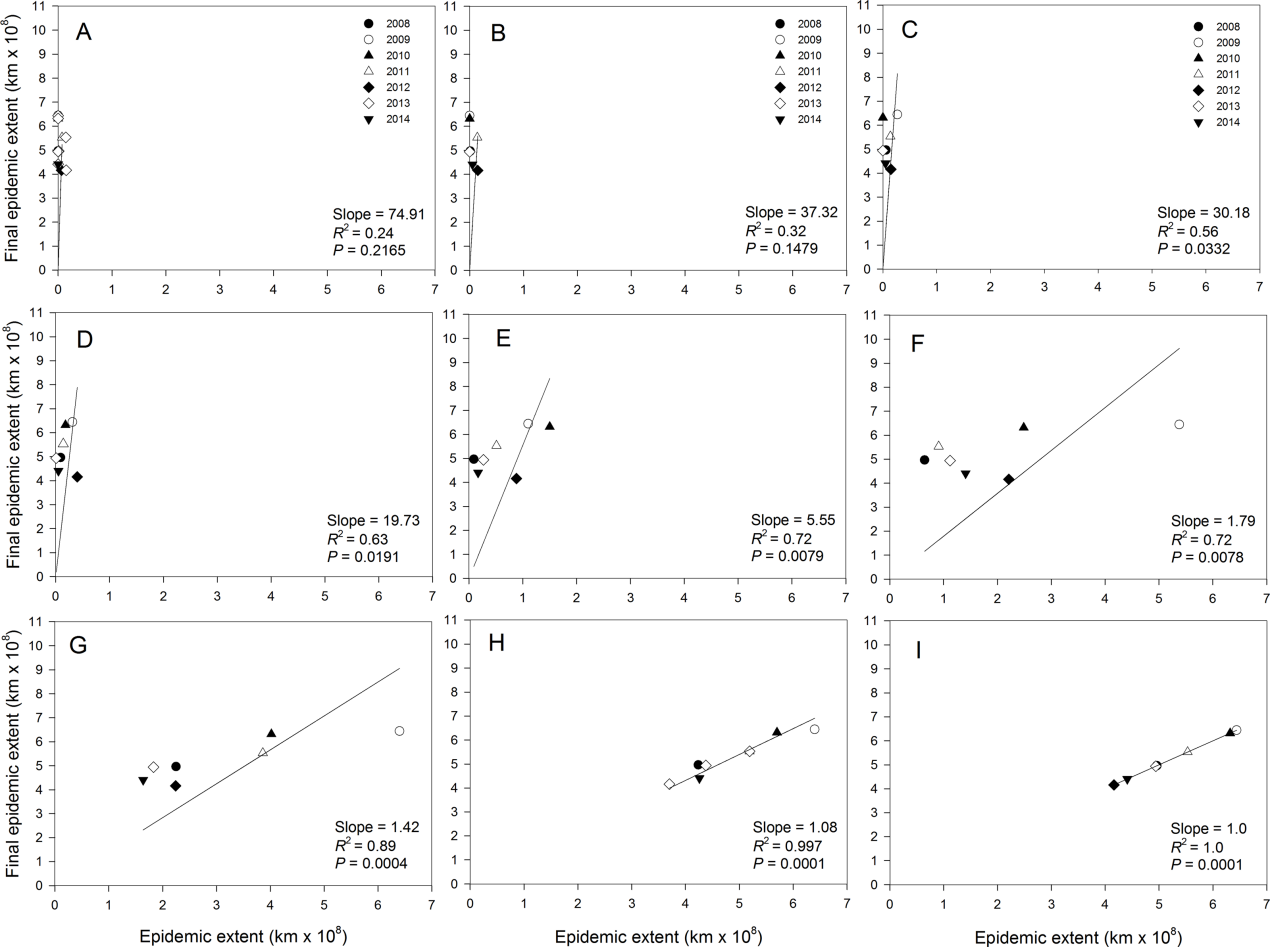
Relationship between final epidemic area and current epidemic area of cucurbit downy mildew. Regression of final epidemic area on epidemicarea of cucurbit downy mildew observed in a week during the same epidemic year. (A) March 31, (B) April 14, (C) April 28, (D) May 14, (E) May 28, (F) June 11, (G) June 25, (H) July 9, and (I) July 23. Final epidemic area is epidemic extent observed on the week ending July 31 for each epidemic year. Note that the regression model is fitted without an intercept.

The strength of association between current and final epidemic extent varied during the course of the epidemics. Regressions of final extent on current epidemic extent showed no association (0.23 ≤ *R*^2^ ≤ 0.31; *P* > 0.1551) from the start of the epidemic in late March (Fig. 4A) to mid-April (Fig. 4B). However, moderate strengths of association were observed from end of April (Fig. 4C) to mid-May (Fig. 4D) with coefficients of determination ranging from 0.56 to 0.63 with significant (*P* < 0.04) regression slopes. The strength of the associations increased in late May to late June with coefficients of determination ranging from 0.72 to 0.89 with highly significant (*P* < 0.0100) regression slopes (Figs. 4E, 4F and 4G). Coefficient of determination during the period of major epidemic expansion from end of May to early July increased from 0.72 to 0.99 and approached 1.0 thereafter until the end of July (Fig. 4I).

### Focus expansion and estimates of spread parameter *b*

**Figure 5:**
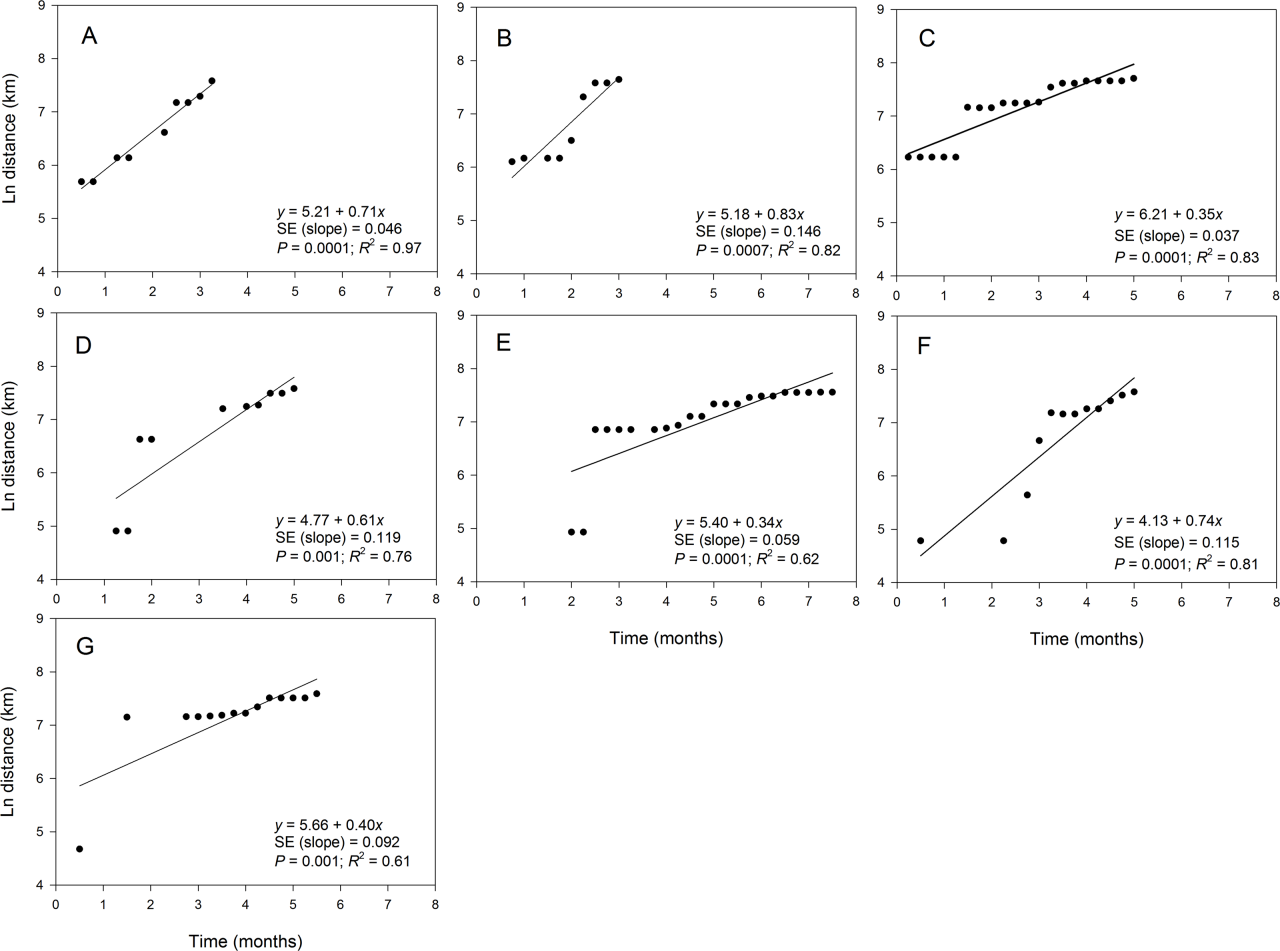
Relationship between the position of epidemic wave-front and time for epidemics examined in specific years. Regression of the position of the epidemicwave-front (log-scale) on time for representative epidemic years. (A) 2008, (B) 2009, (C) 2010, (D) 2011, (E) 2012, (F) 2013, and (G) 2014. Symbols are means of the position of the epidemic wave-front across 10-transect directions.

The maximum distance of the observed disease spread varied slightly among epidemic years and ranged from 1,914 km in 2012 to 2,221 km in 2010 (Table 3). The fit of the regression model of ln(*s*_*t*_) on time varied among epidemic data collected in different years (Fig. 5). For example, coefficients of determination (*R*^2^) for the regression of ln(*s*_*t*_) on time were high (i.e., *R*^2^ > 80%) in 2008 (Fig. 5A), 2009 (Fig. 5B), 2010 (Fig. 5C) and 2013 (Fig. 5F) with coefficients ranging from 0.81 to 0.97 (Table 3). Slopes of the corresponding regression lines ranged from τ = 0.35 to 0.83 (Fig. 5). In contrast, data collected in 2011, 2012 and 2014 were not well described by the regression model of ln(*s*_*t*_) on time as shown by the low *R*^2^ that ranged from 0.61 to 0.76 (Table 3). Slopes of the regression lines for years with poor fit of the regression model were about 0.5-fold lower than those in years with good fit of data to the regression model of ln(*s*_*t*_) on time (Fig. 5).

**Table 3:** Estimates of the spread parameter b of the power law model for dispersal gradients based on temporal and spatial regression analysis of the spread of cucurbit downy mildew in the eastern United States.

Epidemic year	Maximum distance (km)	Temporal regression model^a^					Spatial regression model^b^				
		*R*^2^	*b*	LCL	UCL	*P*-value	*R*^2^	*b*	LCL	UCL	*P*-value
2008	1,959	0.97	1.96	1.79	2.21	0.0001	0.98	1.61	1.34	2.02	0.0005
2009	2,090	0.82	1.77	1.45	2.59	0.0007	0.96	1.51	0.94	3.95	0.0200
2010	2,221	0.83	3.36	2.85	4.15	0.0010	0.59	4.16	−26.69	1.93	0.0742
2011	1,957	0.76	2.20	1.70	3.55	0.0001	0.90	2.62	1.80	4.83	0.0037
2012	1,914	0.62	3.50	2.72	5.22	0.0001	0.95	2.32	1.77	3.35	0.0008
2013	1,953	0.81	1.90	1.58	2.60	0.0001	0.89	2.51	1.67	4.99	0.0050
2014	1,975	0.61	3.02	2.21	5.52	0.0010	0.78	3.75	2.16	14.01	0.0193
Overall mean^c^			2.53					2.64			
Mean of years with good fit^d^			2.24 (2.91)				2.11 (3.96)			

**Notes.**

a The parameter *b* is exponent of the inverse power law model. For temporal regression model, *b* was estimated as: *e*^*τ*^∕(*e*^*τ*^ − 1), where *τ* is the slope of the regression of ln(distance) on time.

b The parameter *b* was estimated as the inverse of slope Ψ in the regression of velocity on distance. LCL and UCL are 95% lower and upper confidence limits of *b*, respectively, back-calculated from standard errors of least squares regression slopes; *R*^2^ denotes the coefficient of determination.

c Denotes the mean of estimates of *b* across all the seven epidemic years.

d Denotes the mean of *b* across years where the data were well described (*R*^2^ ≥ 0.80) by the power law model. Value in parenthesis is the mean of *b* across years where the data were poorly described (*R*^2^ < 0.80) by the power law model.

**Figure 6:**
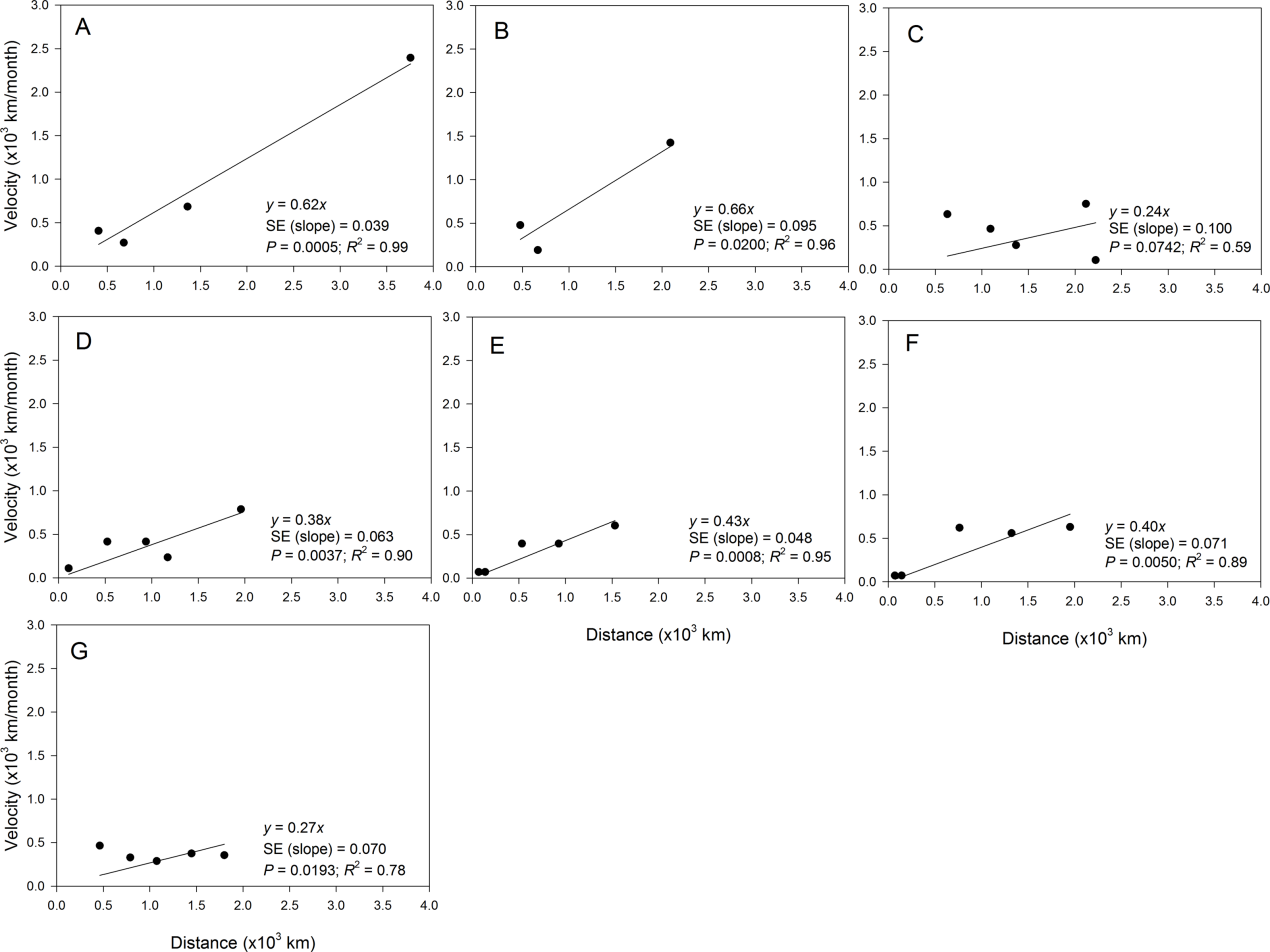
Relationship between the position of epidemic wave-front and distance for epidemics examined in specific years. Regression of the velocity of the epidemic wave-front on distance for representative epidemic years. (A) 2008, (B) 2009, (C) 2010, (D) 2011, (E) 2012, (F) 2013, and (G) 2014. Symbols are means of the position of the epidemic wave-front across 10-transect directions.

The fit of epidemic data to the regression model of *ν*_*t*_ on *s*_*t*_ also varied among epidemic years (Fig. 6) but resulted in a good fit (i.e., *R*^2^ ≥ 0.80) for data collected in all years except in 2010 and 2014 (Table 3). Coefficients of determination in years with good fit of the data to the model ranged from 0.89 to 0.98, while *R*^2^ in years with poor fit for the data ranged from 0.59 to 0.78 (Table 3). However, the slopes of the regression lines varied among all epidemic years. For example, there was about a 2.5-fold difference in slopes for years with good fit to the data to the regression model with slopes ranging from *ψ* = 0.27 in 2014 (Fig. 6G) to *ψ* = 0.66 in 2009 (Fig. 6B). The slope for data collected in 2010 (Fig. 6C) with a poor fit to the regression of *ν*_*t*_ on *s*_*t*_ was considerably lower with *ψ* = 0.24.

Estimates of *b* from the slopes of temporal regression model varied among epidemic years (Table 3). For example *b* ranged from 1.77 in 2009 to 3.50 in 2012. In addition, the 95% confidence limits of *b* contained a value of 2 for epidemics observed in all years except in 2010, 2012 and 2014 (Table 3). The estimate of *b* averaged over years with good fit to the temporal regression model was 2.24, while *b* averaged over years with poor fit to the data was substantially higher with *b* = 2.91. The estimate of *b* averaged over all epidemic years was 2.53 (Table 3). Values of *b* estimated from spatial model ranged from 1.51 in 2009 to 4.16 in 2010. For the temporal regression model, the 95% confidence limits of *b* from the spatial regression model contained a value of 2 for epidemics observed in all years except in 2010 and 2014 (Table 3). The estimate of *b* averaged over years with a good fit to the spatial model was 2.11, which was lower than the average of *b* across all epidemic years (*b* = 2.64) or the average over years with poor fit to the data which (*b* = 3.96) (Table 2).

Covariance analysis conducted for all epidemic years showed that the parameter *b* was significantly (*P* < 0.0015) affected by year and time (or distance) irrespective of the regression model used to estimate *b* except for the spatial model for which *b* was not significantly (*P* = 0.1274) affected by year (Table 4). In addition, the parameter *b* was also significantly (*P* < 0.0015) affected by the interaction between year and time (or distance) when the analysis was performed for all epidemic years (Table 4). These results followed a similar pattern when the analysis was conducted only for years with a good fit to regression models, with the interaction between year and time (or distance) being highly significant (*P* = 0.0001) for the temporal regression or marginally non-significant (*P* = 0.0522) for the spatial regression model (Table 4).

**Table 4:** Analysis of covariance to test the stability ofthe spread parameter the power law model for dispersal gradients based ontemporal and spatial regression of the spread of cucurbit downy mildew in the eastern United States.

	Model used to estimate parameter^b^							
	Temporal regression				Spatial regression			
Analysis^a^	Source	DF	*F*-value	*Pr* > *F*	Source	DF	*F*-value	*Pr* > *F*
I	Year (Y)	6	6.02	0.0001	Year (Y)	7	1.91	0.1274
	Time (T)^b^	1	186.25	0.0001	Distance (D)	1	35.01	0.0001
	Y × T	6	4.54	0.0005	Y × D	6	6.12	0.0012
II	Year (Y)	3	16.71	0.0001	Year (Y)	5	0.45	0.8021
	Time (T)^b^	1	169.63	0.0001	Distance (D)	1	59.92	0.0001
	Y × T	3	9.36	0.0001	Y × D	4	3.09	0.0522

**Notes.**

a Analysis I is based on disease data collected in all epidemic years. Analysis II is based on data in years where the data were well described by the power law model. The model best described the data in 2008, 2009, 2010 and 2013 for the temporal regression, and in 2008, 2009, 2011, 2012 and 2013 for the spatial regression (see Table 2).

b Time is measured on a monthly scale, i.e., in 4-week intervals.

## Discussion

An inverse distance square is proposed as a reasonable approximation for long-distance dispersal described by a power law function for aerially dispersed organisms and other processes that involve dilution over space in turbulent air flow. We tested the stability of the parameter *b* over multiple realizations of an annual northward invasion of cucurbit downy mildew. The disease data sets and conditions associated with spread of epidemics analyzed in this study can be considered typical of many introductions of invasive organisms where the range of the organism is limited by over-seasoning conditions, host occurrence is patchy in the landscape, and resource limitations result in relatively sparse sampling. Under these conditions, we found that estimates of *b* may vary substantially depending on the year and epidemic conditions, ranging from *b* = 1.51 to 4.16. In the temporal regressions, the 95% confidence intervals for *b* included 2 in four of the seven years. In the spatial regressions, the confidence intervals of *b* included 2 in five of the seven years.

The status of disease outbreak in the sentinel plots is usually based on weekly monitoring of the disease until visible symptoms are observed in the plots. Inability to monitor fields regularly coupled with lack of accurate records of when symptoms were first observed can potentially result in measurements errors. Thus, it is probable that true values of *b* are somewhat larger than those reported here due to negatively biased estimates of the slope (Neter, Wasserman & Kutner, 1983). Further, it is reasonable to question the validity of estimates of *b* when the fit of a regression model was marginal, as in 2012 and 2014 with the temporal regressions and 2010 with the spatial regression. In these years, there was poor precision in the estimates of *b*, as indicated by the width of the confidence intervals. When these years are excluded, the overall mean estimated *b* was 2.24 for the temporal regressions and 2.11 in the spatial regressions. Nonetheless, there was still a significant year by time (or distance) interaction, which suggests that *b* varied across epidemic years even when data were well described by the power law model. Variability in estimates of *b* are likely due to numerous factors, namely the shape and distribution of initial source infection, patchy distribution of the host, anisotropic dispersal due to physical barriers such mountain ranges, sampling error, strain-specific interactions between the pathogen and hosts, and the degree of susceptible hosts in the initial outbreak area (Severns et al., 2014). These factors are undoubtedly present in the data sets analyzed here, making interpretation of the stability of *b* “muddy” (Gregory, 1968; Waggoner, 1962). Nonetheless, these factors are common with nearly all invasive (and endemic) organisms and therefore are typical of biological data sets of ecological significance.

Power law relationships in ecology often are empirical and seldom derive from a process-based understanding (Schneider, 2001; Urban, 2005). We note that a parameter estimate of *b* = 2 may provide a useful description of some observed dispersal (disease) gradients but this does not necessarily suggest a mechanistic reason for this process. Complex dispersal patterns caused by human-mediated dispersal, atmospheric conditions during transport, unfavorable environmental conditions, seasonal limitations in host availability, and extreme landscape variability may cause deviations from expected values of *b* (Mundt et al., 2009a; Mundt et al., 2009b). Therefore, the appropriate value of *b* is situation-dependent and more complex spatially and temporally explicit models are needed to describe dispersal when landscape physiognomy and connectivity varies substantially (Brooks, Antonovics & Keitt, 2008; Dunning et al., 1995; Urban, 2005). In the present analyses, parameter estimates of *b* were not stable over time but varied approximately 2-fold among years. The range of values of *b* estimated here could effectively double predicted epidemic velocity depending on which year’s parameter estimates were selected. In these data sets, *b* = 2 might be considered a lower limit of *b*, where year-specific effects (that remain uncertain) may slow epidemic advance. Chatterjee & Dey (2016) describe isotropic dispersal through vertices of a network on a two-dimensional lattice and the appearance of multiple transitions in the speed of spread, with the diameter of the infected area growing according to a function of ∥*s*_1_ − *s*_2_∥^*α*^. Of particular relevance, growth of the infected area is stretched exponentially fast for values of α ranging from 2 to 4 (Chatterjee & Dey, 2016), broadly consistent with estimates of *b* from both the temporal and spatial regressions here and dispersal parameters common across ecological systems.

For pathogens capable of long-distance dispersal described by an accelerating wave, there is an expectation for final epidemic extent to be a multiplicative function of the initial area affected. Over 7 years of observations, we found evidence of this relationship with the cucurbit downy mildew data sets. As noted previously, the correlation between current epidemic extent and final epidemic extent must become more correlated over time as the former variable approaches the latter (Mundt et al., 2013). What is striking in this data set, however, is that there is significant correlative relationship between current and final epidemic area as early as late April, well before cucurbit crops in northern latitude are even planted. The importance of initial disease levels to final epidemic magnitude is exemplified with other plant pathogens at multiple spatial scales, ranging from individual plants (Farber, Medlock & Mundt, 2017), to small and large-scale field plots (Mundt & Sackett, 2012), and at the continental level (Mundt et al., 2013). Simulation studies also suggest an important relationship between small changes in initial outbreak conditions and later rate of disease increase (Severns, Sackett & Mundt, 2015; Xu & Ridout, 1998). More broadly, propagule pressure is closely linked to the likelihood of successful establishment of an organism in a new habitat, overcoming geographic, physical, and biological factors that may normally prevent invasion by a species (Simberloff, 2009). In the present analyses, it is salient that this relationship exists at the continental scale despite varying environmental conditions, management intervention, and area planted to the host. This suggests that conditions of the initial outbreak are a primary determinant of epidemic spread and outbreak magnitude, a conclusion supported by a growing number of studies in multiple pathosystems (Estep, Sackett & Mundt, 2014; Mundt et al., 2013; Severns et al., 2014; Smith et al., 1988).

Cucurbit downy mildew is active and commonly detected after July. In present study, the end of July marked the termination of the temporal extent of the epidemic analysis. We intentionally restricted analyses from March to July to avoid potential confounding from later planted cucurbit crops. In the southern US and mid-Atlantic region there are often two plantings of cucurbits, the second beginning in mid to late summer. Attempts to estimate the initial or current epidemic area when multiple, overlapping generations of the host are planted would be impossible with the present data. An important assumption in this analysis is that final epidemic extent should be 0 when initial extent is also 0, i.e., that the regression should be fit without an intercept. While the present analyses have attempted to minimize errors in estimation of initial epidemic area by restricting the analysis to the first planting of cucurbits, there is still a large uncertainty in the initial epidemic area. In reality, the initial epidemic area is always non-zero because cucurbit downy mildew is active year-round in areas along the Gulf of Mexico (Ojiambo et al., 2015). A similar uncertainty also exists for soybean rust (Christiano & Scherm, 2007; Mundt et al., 2013) and other temperature-sensitive organisms that over-winter in subtropical climates. It is difficult to know the impact of this error in this study. We do note, however, that the fit of the relationship at the continental scale, though significant, was suboptimal for certain time points (i.e., 28 May and 11 June in Fig. 4) and would largely disappear if an intercept term was fit. Nonetheless, the relationship still provides a rough approximation of the potential for epidemic extent based on characteristics of early epidemic conditions. Of course, the methods described here provide only a description of the generalized epidemic wave-front and magnitude but provide no information about the disease risk of individual sentinel plots or counties. Disease risk at these scales requires more detailed information on precise weather conditions at the scale of crop canopy and long-distance transport and deposition of inoculum from mechanistic models (Arauz et al., 2010; Neufeld, Isard & Ojiambo, 2013; Ojiambo & Holmes, 2011). Nonetheless, the findings here suggest that the magnitude of epidemics at the landscape level may be largely determined by antecedents associated with overwintering success of the pathogen in southern latitudes.

##  Supplemental Information

10.7717/peerj.3465/supp-1 Supplemental Information 1 Datasets for current and final extent, prevalence, and velocity

## Additional Information and Declarations

### Data Availability

The following information was supplied regarding data availability: The raw data has been supplied as a Supplementary File.
